# Long COVID as a network disorder: a mechanism-anchored framework for biological stratification and therapeutic targeting

**DOI:** 10.3389/fmed.2026.1841690

**Published:** 2026-05-29

**Authors:** Robert Groysman

**Affiliations:** Independent Researcher, Plano, TX, United States

**Keywords:** biological heterogeneity, dysautonomia, endothelial dysfunction, Long COVID, mechanistic stratification, mitochondrial dysfunction, network disorder, post-acute sequelae of SARS-CoV-2 infection

## Abstract

Long COVID is increasingly recognized as a biologically heterogeneous, multisystem condition with wide variability in symptom expression and treatment response. Although symptom-based phenotyping has advanced descriptive and epidemiologic understanding, similarity in clinical presentation does not necessarily imply shared upstream pathophysiology. Therapeutic cohorts defined solely by symptom clusters may therefore combine biologically distinct mechanisms, potentially diluting treatment effects and complicating interpretation of interventional trials. This manuscript proposes a complementary, mechanism-anchored framework centered on recurrent and biologically measurable domains: autonomic dysfunction, mitochondrial and bioenergetic impairment, endothelial and microvascular dysfunction, gut dysbiosis and barrier disruption, mast cell–mediated signaling, and neuroendocrine dysregulation. These primary domains are conceptualized as physiologically coherent systems capable of generating multisystem symptom patterns in biologically enriched subsets. Secondary amplifying processes, including persistent immune activation, viral antigen persistence without established replication, autoantibody formation, neuroinflammation, and sleep-related destabilization, are positioned as network-coupling processes that may sustain or amplify dysregulation across domains. Within a network-based framework, Long COVID is conceptualized as a network disorder in which interacting regulatory nodes are hypothesized to generate self-reinforcing feedback loops that maintain symptom persistence even after resolution of acute infection. The model accommodates heterogeneity across mechanisms and alternative explanatory hypotheses. It provides an operational structure for organizing mechanistic heterogeneity and generating testable predictions. A prototype, unvalidated screening instrument is included to illustrate a potential pathway toward mechanism-informed stratification and prospective trial enrichment. Future research should evaluate whether biologically enriched cohorts demonstrate differential therapeutic responsiveness compared with symptom-defined populations. Prospective validation using standardized physiological metrics will be essential to determine whether mechanism-guided stratification improves translational precision and clarifies actionable pathophysiology in Long COVID.

## 1 Introduction

Long COVID, also referred to as post-acute sequelae of SARS-CoV-2 infection, is characterized by persistent or relapsing symptoms following acute infection and affects a substantial proportion of individuals in reported cohorts (1, 2). Clinical presentations are heterogeneous and may include fatigue, cognitive dysfunction, orthostatic intolerance, dyspnea, gastrointestinal disturbances, neuropathic symptoms, and endocrine abnormalities (1, 2).

Although the risk of Long COVID is higher after severe acute COVID-19 and hospitalization, persistent post-COVID symptoms have also been documented after non-hospitalized and initially mild infections, and the large absolute number of mild infections contributes substantially to the overall Long COVID burden (3–7, 56). Acute COVID-19 has been associated with widespread inflammatory activation, vascular injury, and multiorgan involvement across respiratory, cardiovascular, neurologic, and metabolic systems, suggesting potential pathways through which post-acute physiological disturbances may emerge (8).

Efforts to characterize this heterogeneity have largely relied on symptom-based phenotyping. Large cohort analyses and machine-learning approaches have identified consistent symptom clusters, including cardiopulmonary, cognitive, fatigue-dominant, and multisystem patterns (3, 4, 9, 10). These approaches have improved descriptive classification and epidemiologic understanding. However, similarity in symptom presentation does not necessarily imply shared upstream biological mechanisms.

Emerging research suggests physiological heterogeneity in Long COVID. Studies across immunology, metabolomics, vascular biology, and autonomic testing have identified diverse abnormalities in subsets of patients, including autonomic dysfunction and postural orthostatic tachycardia syndrome (11, 12), exertion-related metabolic impairment and mitochondrial dysfunction (13), endothelial and microvascular abnormalities (8), gut microbiome alterations (14), mast cell–associated signaling in selected cohorts (15–18), and disturbances in neuroendocrine regulation (19–23). Additional processes including persistent immune activation (24, 25), tissue viral antigen persistence (26, 27), autoantibody formation (28), neuroinflammatory changes suggested by imaging and experimental models (29, 30), and small fiber neuropathy (31) have also been reported in subsets of individuals.

These observations highlight the biological heterogeneity of Long COVID, involving multiple interacting physiological systems rather than a single uniform post-viral process. In complex diseases characterized by multisystem involvement, disruption of interconnected regulatory networks can produce persistent symptoms even after the initiating trigger has resolved (32).

This manuscript proposes a mechanism-anchored conceptual framework that distinguishes primary mechanistic domains from secondary amplifying processes and interprets Long COVID as a network disorder sustained by interacting physiological systems. This framework complements symptom phenotyping by examining whether stratification anchored in measurable biology may improve mechanistic clarity and generate testable hypotheses for therapeutic targeting and clinical trial enrichment. This framework provides an operational structure that explicitly links measurable physiological domains with stratification and testable clinical predictions.

## 2 Symptom-based phenotyping and its limitations

### 2.1 Emergence of symptom-defined clusters

Symptom-based phenotyping has played a central role in characterizing the clinical heterogeneity of Long COVID. Large cohort analyses using unsupervised machine-learning methods, digital health tracking platforms, and large-scale survey datasets have identified consistent symptom clusters across populations (3, 4, 9, 10). These clusters commonly include cardiopulmonary symptoms, cognitive dysfunction, fatigue-dominant patterns, and multisystem presentations.

Such studies have provided important descriptive insight into the variability of post-acute symptoms and have facilitated hypothesis generation regarding potential subgrouping. Large research initiatives, including the RECOVER program and related national cohort studies, have further advanced efforts to characterize symptom trajectories and identify predictors of prolonged recovery (4). In parallel, patient-reported outcome analyses have attempted to evaluate perceived treatment responses across symptom-defined populations (10). While valuable for descriptive and epidemiologic purposes, these approaches primarily capture patterns of symptom co-occurrence rather than underlying biological mechanisms.

Collectively, these studies highlight the clinical diversity of Long COVID and underscore the importance of structured classification systems for descriptive epidemiology.

### 2.2 Limitations of symptom-defined treatment stratification

Although symptom clustering provides important descriptive insight, symptoms represent downstream manifestations of upstream physiological processes. Shared symptom profiles do not necessarily reflect shared mechanisms.

For example, cognitive impairment in Long COVID has been associated with autonomic dysfunction and orthostatic intolerance (11, 12), neuroinflammatory processes suggested by imaging and experimental models (29, 30), exertion-related metabolic abnormalities (13), and vascular or endothelial disturbances (8). These mechanisms may overlap within individual patients but are not biologically equivalent.

Similarly, fatigue—a hallmark symptom of Long COVID—has been linked to dysautonomia (11, 12), mitochondrial and metabolic inefficiency (13), inflammatory signaling (24, 25), endocrine dysregulation (19–23), and vascular dysfunction (8). The relative contribution of these mechanisms likely varies across individuals and cohorts.

When treatment cohorts are defined solely by symptom similarity, biologically distinct subgroups may be combined within a single interventional framework. In heterogeneous network disorders, such mechanistic diversity may attenuate detectable treatment effects and complicate interpretation of therapeutic responses (25, 32).

Consistent with this concern, interventional studies conducted in broadly defined Long COVID populations have reported mixed or modest therapeutic benefits (33, 34). Adaptive platform trials such as STIMULATE-ICP aim to evaluate multiple therapeutic strategies within heterogeneous cohorts (35), yet biological diversity within enrolled populations may continue to influence treatment responsiveness.

These observations suggest that symptom similarity alone may be insufficient for therapeutic stratification and highlight the potential importance of approaches anchored in measurable physiological mechanisms.

### 2.3 Phenotypes versus endotypes

The distinction between clinical phenotypes and biologically defined endotypes is well established in other heterogeneous chronic diseases. In airway disease, identification of biologically defined endotypes improved therapeutic targeting beyond symptom classification alone by linking clinical presentation to measurable pathophysiology (36, 37).

A similar conceptual distinction may be relevant in Long COVID. Symptom phenotypes describe observable clinical patterns, whereas mechanistic domains correspond to biologically defined endotypes reflecting upstream physiological disturbances that may be more directly measurable and potentially targetable.

Integration of mechanistic assessment with symptom characterization may improve biological coherence within treatment cohorts and enhance interpretability of therapeutic outcomes.

### 2.4 Network medicine and mechanistic evidence

Network-based models of disease propose that chronic illness may arise not from a single isolated pathway but from dysregulation across interacting biological systems (32). In this framework, immune signaling, autonomic regulation, vascular function, metabolism, endocrine signaling, and gut barrier integrity can be understood as interconnected regulatory nodes capable of influencing one another through bidirectional feedback loops. Perturbation within one system may therefore propagate across others, generating self-reinforcing physiological instability even after the initiating trigger has diminished.

This systems-based perspective also intersects with emerging discussions regarding mechanistic evidence in medicine. Although traditional evidence hierarchies have often prioritized randomized controlled trials, mechanistic evidence may provide important complementary value in heterogeneous multisystem disease, particularly when biologically distinct subgroups may respond differently to treatment (38–40). Importantly, mechanisms do not need to be fully mapped in every intermediate step to provide useful explanatory or stratifying value. The present framework therefore does not assume complete mechanistic resolution of Long COVID biology, but proposes an operational structure for organizing recurrent physiological patterns into testable mechanistic domains. In this context, the present manuscript proposes an operational approach for organizing mechanistic heterogeneity in Long COVID using biologically measurable physiological domains.

## 3 Operational definition of primary mechanistic domains and secondary amplifiers

### 3.1 Defining primary mechanistic domains

Long COVID is increasingly recognized as a biologically heterogeneous condition involving interacting disturbances across immunologic, vascular, neurologic, metabolic, and endocrine systems (8, 24, 25). To organize this heterogeneity for translational purposes, this manuscript defines primary mechanistic domains using explicit operational criteria.

A primary mechanistic domain is defined here as a biological process that satisfies several characteristics:

1. Recurrent observation across independent cohorts.
  The domain should be reported across multiple studies using different investigative methodologies.
2. Capacity to generate multisystem symptom expression.
  The mechanism should plausibly contribute to symptoms affecting multiple physiological systems, consistent with the multisystem nature of Long COVID (1, 8).
3. Objective measurability.
  The domain should be assessable using established physiological testing, laboratory markers, imaging techniques, functional assessments, or validated clinical criteria. However, objective measurability does not imply uniform standardization across methodologies or clinical settings. Variability in acquisition protocols, assay sensitivity, biomarker thresholds, and reproducibility may influence domain assignment, cohort enrichment, and cross-study comparability. Accordingly, future validation of mechanism-based stratification will require increasingly standardized and reproducible physiological assessment frameworks.
4. Direct clinical targetability.
  The domain should represent a physiological process that can be directly targeted at the systems level, rather than a fixed trait or background predisposition.
5. Participation in cross-system feedback interactions.
  The mechanism should plausibly interact with other physiological systems in a manner that allows dysregulation to persist beyond the initiating trigger, consistent with principles of network medicine in complex disease states (32). These criteria provide a structured approach without imposing strict exclusivity or hierarchy.
6. Importantly, classification of a domain as “primary” within this framework does not imply universal temporal primacy or exclusive causal initiation. In complex network disorders, physiological domains may function simultaneously as initiating contributors, downstream consequences, and feedback-mediated amplifiers depending on individual patient context and disease stage. In many cases, causal sequencing may remain indeterminate and requires prospective longitudinal investigation. These criteria help distinguish upstream biological organization from downstream symptom expression.

Using these criteria, six primary mechanistic domains are proposed:

1. Dysautonomia, including sympathetic overactivation, postural orthostatic tachycardia syndrome, and vagal dysfunction (11, 12)
2. Mitochondrial dysfunction and impaired bioenergetics, including post-exertional symptom exacerbation phenotypes (13)
3. Endothelial and microvascular dysfunction, including immunothrombotic features (8)
4. Gut dysbiosis and barrier dysfunction (14, 41–43)
5. Mast cell activation and histamine-mediated signaling (15–18)
6. Neuroendocrine and sex hormone dysregulation (19–23)

Each of these domains has been described in peer-reviewed Long COVID cohorts, although prevalence and relative contribution vary across studies (8, 11–14, 19, 24–26, 28, 29, 31). Domains may overlap within individual patients. In some cases, one domain may appear clinically predominant, whereas in others multiple domains may act in parallel.

The framework therefore allows for both domain predominance and multidomain coupling.

These domains are intended as operational and heuristic constructs for translational stratification rather than fixed or mutually exclusive biological entities.

### 3.2 Defining secondary amplifying mechanisms

In addition to primary mechanistic domains, several biological processes have been described that may amplify, sustain, or couple dysregulation across domains in subsets of individuals with Long COVID.

A secondary amplifying mechanism is defined here as a biological process that arises downstream of primary domains, propagates dysregulation across systems, and interacts bidirectionally with other components of the network.

Such mechanisms typically:

- Arise downstream of one or more primary mechanistic domains.
- Intensify or propagate dysregulation across physiological systems.
- Frequently interact bidirectionally with primary domains.

These mechanisms are not necessarily sufficient to generate the full clinical syndrome and may be present in subsets rather than universally.

- Examples described in Long COVID cohorts include:
- Persistent immune activation and inflammatory signaling (24, 25).
- Viral antigen persistence in tissue reservoirs in selected studies (26, 27).
- Autoantibody formation affecting receptor signaling in subsets of patients (28).
- Neuroinflammation and microglial activation suggested by imaging and experimental models (29, 30).
- Small fiber neuropathy affecting autonomic and sensory fibers (31).

These processes are not secondary in importance but are conceptualized as amplifiers capable of strengthening feedback loops that sustain physiological dysregulation.

For example:

- Persistent immune activation may exacerbate endothelial dysfunction and autonomic instability (24, 25).
- Small fiber neuropathy may reinforce dysautonomia through structural autonomic fiber impairment (31).
- Viral antigen persistence, where present, may sustain inflammatory signaling through continued antigen exposure (26, 27).
- Autoantibody formation may alter receptor signaling affecting autonomic, vascular, or endocrine pathways (28).

Positioning these mechanisms as amplifiers rather than universal primary drivers reflects their variable presence across cohorts. Their presence varies across cohorts, and causal sequencing remains incompletely defined.

Such processes may contribute to symptom persistence by reinforcing cross-domain coupling and physiological instability (32).

### 3.3 Long COVID as a network disorder

The distinction between primary mechanistic domains and secondary amplifying processes aligns with principles of network-based models of complex disease. In such models, chronic illness does not arise from a single upstream causal pathway but from interacting biological systems whose dysregulation may reinforce one another through bidirectional feedback (32).

Network medicine frameworks propose that disruption of interconnected regulatory systems can generate self-sustaining pathological states, even after the initiating trigger has diminished or resolved. These models have been applied to a range of chronic multisystem disorders characterized by heterogeneous symptoms, fluctuating severity, and variable treatment responsiveness (32).

Long COVID exhibits features consistent with a network-based disease process. Multiple physiological systems implicated in Long COVID, including autonomic regulation, vascular function, immune signaling, metabolic homeostasis, endocrine regulation, and gut barrier integrity, are biologically interconnected. Perturbation within one system can therefore propagate across others through bidirectional physiological interactions.

Examples of potential cross-domain interactions include:

- Autonomic dysregulation altering vascular tone and shear stress, potentially influencing endothelial function (11, 12).
- Endothelial dysfunction impairing tissue perfusion, which may exacerbate metabolic strain and mitochondrial stress (8).
- Mitochondrial dysfunction increasing oxidative stress, thereby amplifying inflammatory signaling pathways (24, 25).
- Inflammatory cytokines modifying neuroendocrine signaling, potentially affecting stress-axis regulation and hormonal balance (19, 24, 25).
- Gut barrier dysfunction enabling microbial translocation, which may sustain immune activation and endothelial inflammation (14, 41–43).

Not all domains are expected to be present in all individuals. Rather, they illustrate how dysregulation in one biological node may propagate through interconnected systems, contributing to persistent symptoms even if viral replication is no longer active.

This network perspective shifts emphasis away from identifying a single universal mechanism and toward identifying dominant physiological nodes within a dynamic system. In individual patients, one domain may exert disproportionate influence over the broader network, with secondary domains emerging downstream through feedback interactions.

Such a framework is consistent with the substantial heterogeneity observed in Long COVID cohorts. It also provides a conceptual basis for mechanism-based stratification strategies in which therapeutic targeting focuses on dominant physiological disturbances rather than symptom similarity alone.

**Figure 1:**
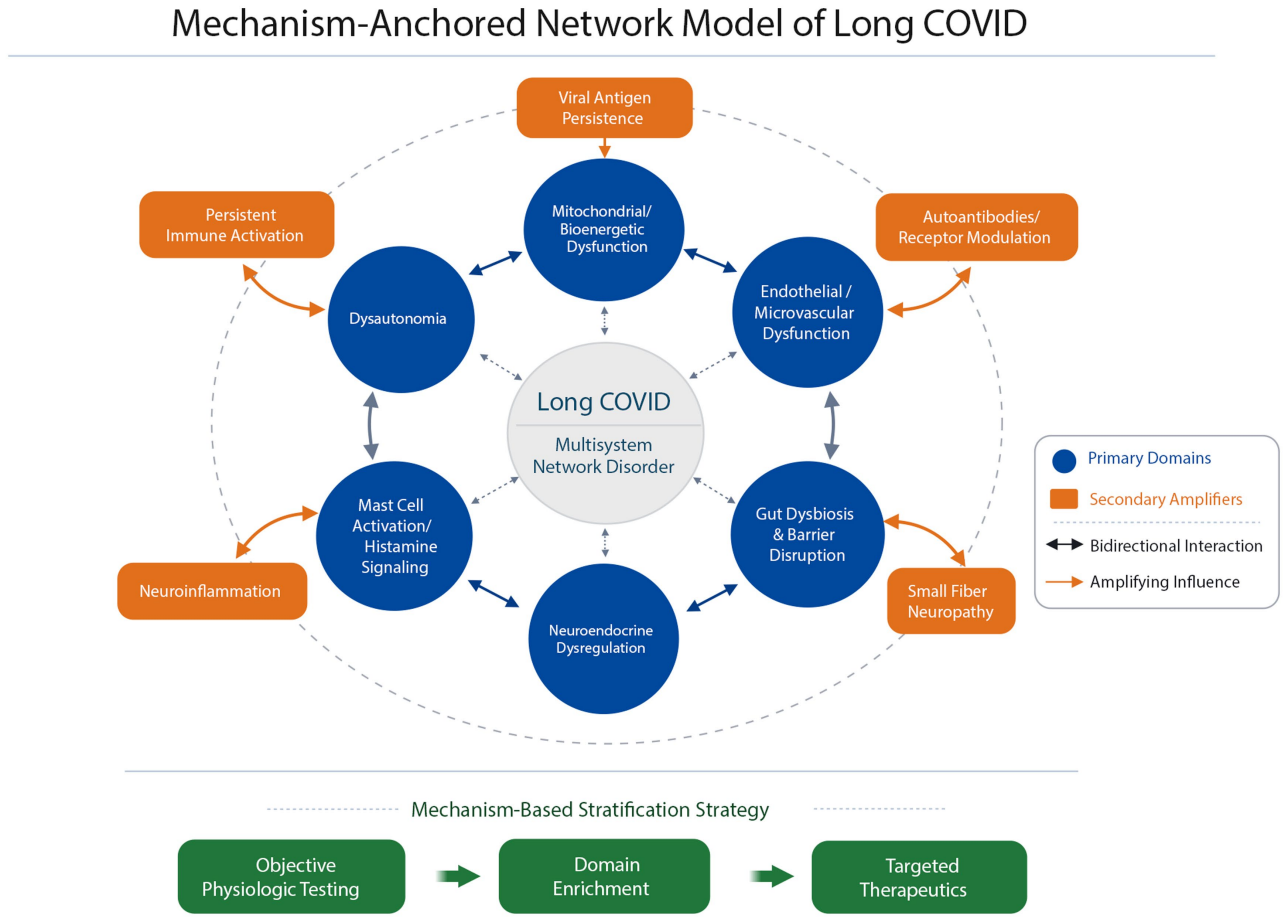
Primary physiological domains are represented as the central network nodes capable of independently generating multisystem symptoms. Secondary processes such as immune activation, autoantibodies, viral antigen persistence, and neuroinflammation function as amplifiers that may reinforce instability within the network rather than acting as universally dominant initiating mechanisms. Illustrated interactions are representative rather than exhaustive.

The following sections examine each proposed primary mechanistic domain in greater detail using the operational criteria described above (Figure 1; Table 1).

**Table 1:** Testable predictions derived from a mechanism-based stratification framework in long COVID primary mechanistic domains represent physiologic regulatory systems capable of independently generating multisystem symptoms, whereas secondary amplifiers represent processes that intensify or sustain dysregulation across domains.

Primary mechanistic domain	Example objective measures	Hypothesis-generating translational prediction
Dysautonomia	Tilt-table testing, active stand test, heart rate variability metrics, plasma catecholamines (11, 12)	Patients with objective orthostatic abnormalities may demonstrate greater responsiveness to autonomic-modulating therapies than symptom-defined cohorts alone
Mitochondrial/bioenergetic dysfunction	Cardiopulmonary exercise testing, exertional lactate dynamics, metabolomic profiling (8, 13)	Patients with exertion-triggered metabolic instability may demonstrate clearer functional improvement with metabolic-targeted or mitochondrial-supportive interventions
Endothelial/microvascular dysfunction	Flow-mediated dilation, endothelial biomarkers, microvascular perfusion imaging (8, 46)	Vascular-directed therapies may demonstrate stronger therapeutic signal in patients with measurable endothelial dysfunction
Gut Dysbiosis/barrier dysfunction	Microbiome profiling, permeability-associated markers, LPS-related biomarkers (14, 41–43)	Gut-targeted interventions may preferentially improve inflammatory and systemic symptoms in biologically enriched subsets
Mast cell activation	Serum tryptase, histamine metabolites, and other mast cell mediator panels, when elevated or obtained during symptomatic periods (15–17)	Histamine blockade or mast cell-modulating therapies may yield differential responses in patients with mediator elevation or classic mast cell symptom patterns
Neuroendocrine dysregulation	Thyroid panel, cortisol rhythm assessment, sex hormone levels (19–23)	Hormonal correction in patients with objective endocrine abnormalities may produce broader multisystem improvement than in unselected cohorts
Secondary amplifiers: immune activation, neuroinflammation, autoantibodies, viral antigen persistence	Cytokine profiling, CSF inflammatory markers, neuroimaging evidence of microglial activation, autoantibody panels, tissue antigen detection in selected cases (24–30)	Combination or multi-domain therapies may demonstrate greater clinical improvement in patients with objective immune activation, neuroinflammation, or viral persistence markers

This table illustrates how domain-based stratification generates falsifiable predictions. The framework is inherently falsifiable: if biologically enriched cohorts do not demonstrate differential treatment responsiveness compared with symptom-defined groups, the model would require refinement. HRV, heart rate variability; LPS, lipopolysaccharide.

Objective measures listed are supported by published physiological or biomarker studies in Long COVID or related post-infectious syndromes. Stratification hypotheses and predicted interventional responses are hypothesis-generating and are intended to guide future prospective validation.

### 3.4 Dysautonomia as a primary mechanistic domain

Autonomic dysfunction has been consistently described in subsets of individuals with Long COVID and represents a commonly reported physiological abnormality following SARS-CoV-2 infection (11, 12). Clinical manifestations may include orthostatic intolerance, tachycardia, palpitations, exercise intolerance, thermoregulatory instability, gastrointestinal dysmotility, and labile blood pressure. Observational cohorts and clinical reports have documented the emergence of postural orthostatic tachycardia syndrome (POTS) and related autonomic disorders following COVID-19 infection (11, 12).

Autonomic testing in post-COVID populations has identified abnormalities in some cohorts in heart rate regulation, blood pressure control, and autonomic balance. Reduced heart rate variability and impaired autonomic flexibility have been reported in some cohorts, consistent with altered sympathetic–parasympathetic regulation (11, 12). Although methodologies vary across studies, converging evidence suggests dysregulation of autonomic signaling in a subset of individuals with Long COVID.

Autonomic regulation influences numerous physiological systems, including vascular tone, gastrointestinal motility, endocrine signaling, immune responses, and metabolic regulation. Dysregulation within this domain therefore has the potential to generate multisystem symptom patterns, consistent with the clinical presentation of Long COVID.

In some individuals, structural autonomic nerve injury may contribute to dysautonomia. Small fiber neuropathy affecting autonomic fibers has been reported in subsets of post-COVID patients and may provide a structural substrate for autonomic dysfunction in certain cases (31). Inflammatory signaling and immune dysregulation may further influence autonomic control through effects on central and peripheral autonomic circuits (24, 25).

#### 3.4.1 Sympathetic overactivation and “fight–flight” dominance

Several studies describe patterns consistent with heightened sympathetic tone in individuals with Long COVID, including persistent tachycardia, palpitations, sleep disturbance, and exaggerated physiological responses to stress (11, 12). Reduced heart rate variability observed in some cohorts suggests diminished parasympathetic buffering and relative sympathetic predominance.

Sustained sympathetic activation can influence multiple downstream systems. Catecholaminergic signaling modulates vascular tone, immune cell trafficking, inflammatory mediator release, and metabolic regulation. Neuroimmune interactions between adrenergic signaling pathways and inflammatory processes have been described in other chronic inflammatory conditions, providing biological plausibility for amplification loops within Long COVID (24, 25).

Sympathetic predominance may therefore interact with endothelial dysfunction and inflammatory signaling, potentially contributing to symptom amplification in biologically susceptible individuals.

#### 3.4.2 Postural orthostatic tachycardia syndrome and orthostatic intolerance

Postural orthostatic tachycardia syndrome (POTS) has been increasingly reported following SARS-CoV-2 infection (12). Diagnostic criteria typically include excessive heart rate increase upon standing without orthostatic hypotension, accompanied by symptoms such as lightheadedness, fatigue, palpitations, exercise intolerance, and cognitive difficulty.

The pathophysiology of post-COVID POTS remains incompletely defined but may involve impaired peripheral vasoconstriction, relative hypovolemia, autonomic receptor dysregulation, or small fiber neuropathy (12, 31). Reduced cerebral perfusion during orthostatic stress provides a plausible physiological mechanism linking autonomic instability with cognitive symptoms frequently reported in Long COVID.

Altered vascular tone and impaired venous return may also interact with endothelial dysfunction and metabolic stress. POTS represents a clinically defined manifestation within the broader dysautonomia domain rather than a separate independent mechanism.

#### 3.4.3 Vagal dysfunction and parasympathetic withdrawal

In addition to sympathetic overactivation, reduced parasympathetic signaling may contribute to autonomic imbalance in Long COVID (11). Decreased vagal tone can impair heart rate variability, gastrointestinal motility, and cholinergic anti-inflammatory signaling.

The vagus nerve plays a central role in the regulation of inflammatory responses through the cholinergic anti-inflammatory pathway. Reduced vagal activity may therefore contribute to sustained inflammatory signaling and impaired autonomic regulation (24, 25).

Autonomic regulation of gastrointestinal motility also links vagal function to the gut–brain axis. Symptoms such as nausea, early satiety, bloating, and altered bowel habits may reflect dysregulation of enteric autonomic signaling, creating bidirectional interactions between dysautonomia and gut dysbiosis (14).

Autonomic dysfunction also interacts with multiple other physiological domains. Dysautonomia may:

- Influence vascular tone and endothelial signaling.
- Affect mitochondrial energy metabolism through perfusion changes.
- Modulate immune responses through neuroimmune pathways.
- Alter gastrointestinal function and microbiome composition.
- Interact with endocrine signaling through stress-axis regulation.

These multidirectional interactions position dysautonomia as a central regulatory node within the proposed network model of Long COVID rather than a peripheral clinical feature.

### 3.5 Mitochondrial dysfunction and bioenergetic impairment

Persistent fatigue, exercise intolerance, and delayed recovery following exertion are among the most frequently reported symptoms in Long COVID (1, 2). Increasing evidence suggests that disturbances in cellular energy metabolism may contribute to these symptoms in a subset of individuals.

Studies of post-COVID populations have identified abnormalities in skeletal muscle metabolism, oxygen extraction, and energy utilization during exertion (13). Altered substrate utilization and impaired recovery following exertional stress have also been described, suggesting disruptions in bioenergetic homeostasis rather than simple deconditioning. In addition, broader mechanistic analyses of Long COVID have highlighted metabolic and mitochondrial pathways as potential contributors to persistent symptoms (8).

Mitochondria play a central role in ATP production, redox balance, apoptotic signaling, and immune–metabolic coupling. Impairment in oxidative phosphorylation, mitochondrial dynamics, or organelle turnover can therefore produce multisystem physiological consequences. Dysregulated mitochondrial function has been associated with altered metabolic signaling, increased oxidative stress, and inflammatory amplification in a variety of chronic conditions, providing biological plausibility for similar processes in Long COVID.

In addition to reduced energy production, disturbances in mitochondrial quality control mechanisms may contribute to persistent metabolic instability. Mitophagy and broader autophagic pathways remove damaged mitochondria and maintain cellular redox balance. Disruption of these pathways may allow accumulation of dysfunctional mitochondria, promoting reactive oxygen species generation and inflammatory signaling (44, 45). Although direct longitudinal evidence in Long COVID remains limited, impairment of mitochondrial quality control represents a plausible mechanism linking infection-related cellular stress to persistent bioenergetic dysfunction.

#### 3.5.1 Bioenergetic impairment without post-exertional malaise

Some individuals report persistent fatigue and reduced exercise tolerance without delayed symptom worsening following activity. In these cases, metabolic inefficiency may arise from impaired oxidative phosphorylation, endothelial-mediated limitations in oxygen delivery, or chronic low-grade inflammatory signaling (8, 13, 24).

Inflammatory cytokines can alter mitochondrial metabolism and shift cellular energy production toward less efficient pathways, linking immune activation with metabolic stress (24, 25). Endothelial dysfunction may further exacerbate tissue-level hypoxia and oxidative stress by impairing microvascular perfusion (8). Together, these interactions provide a biologically plausible explanation for persistent fatigue in individuals without classic post-exertional symptom exacerbation.

#### 3.5.2 Post-exertional malaise–associated instability

A subset of individuals with Long COVID experience post-exertional malaise (PEM), characterized by delayed symptom worsening following physical or cognitive exertion. In such cases, even modest activity may trigger symptom exacerbation hours to days after exertion.

Recent investigations have reported skeletal muscle abnormalities that worsen following exertional stress in individuals with Long COVID, supporting the concept of impaired metabolic recovery following activity (13). Proposed mechanisms include exaggerated oxidative stress responses, impaired restoration of redox balance, altered mitochondrial dynamics, and instability in mitochondrial quality control pathways (44, 45).

The delayed onset of PEM suggests impaired restoration of metabolic homeostasis rather than simple reduction in baseline energy production. PEM may reflect instability in bioenergetic regulation following physiological stress.

Mitochondrial dysfunction also interacts with multiple physiological domains. For example:

- Inflammatory signaling may impair mitochondrial metabolism (24, 25).
- Endothelial dysfunction may reduce oxygen delivery and exacerbate metabolic stress (8).
- Endocrine disturbances may alter mitochondrial biogenesis and substrate utilization (19).

These multidirectional interactions position mitochondrial dysfunction as a central interacting domain rather than an isolated metabolic abnormality.

### 3.6 Endothelial and microvascular dysfunction

Vascular and endothelial abnormalities were prominent features of acute SARS-CoV-2 infection and remain biologically relevant in post-acute disease states (8). Endothelial cells regulate vascular tone, nitric oxide bioavailability, coagulation balance, and inflammatory signaling. Persistent endothelial activation or dysfunction may therefore contribute to multisystem symptom expression in subsets of individuals with Long COVID.

#### 3.6.1 Endothelial activation and impaired vascular regulation

Endothelial dysfunction has been reported in post-COVID cohorts, including abnormalities in vascular reactivity and markers of vascular inflammation (8). Impaired nitric oxide signaling and altered microvascular responsiveness may contribute to dysregulated tissue perfusion.

Reduced perfusion may exacerbate metabolic strain and contribute to exertional intolerance. Impaired cerebral microvascular regulation also provides a plausible mechanism linking vascular dysfunction with cognitive symptoms observed in Long COVID. In addition, autonomic instability may influence vascular tone and shear stress, creating bidirectional interactions between dysautonomia and endothelial function (11, 12).

Persistent immune activation may further amplify endothelial dysfunction through cytokine-mediated signaling pathways (24, 25), creating coupling between inflammatory and vascular domains.

#### 3.6.2 Immunothrombosis and microvascular fibrin pathology

Alterations in coagulation signaling and fibrinolytic balance have been described in subsets of individuals with Long COVID (8). Reports of fibrin amyloid microclot formation and impaired fibrinolysis have generated substantial interest as potential contributors to microvascular dysfunction (46).

However, detection methodologies and prevalence estimates vary across studies, and reproducibility across cohorts remains an area of active investigation. Microclot phenomena are therefore considered one potential manifestation of broader endothelial and immunothrombotic dysregulation rather than an isolated primary mechanism.

Microvascular obstruction or altered rheological properties could impair oxygen delivery and tissue perfusion, thereby contributing to metabolic stress and bioenergetic dysfunction. Such interactions illustrate how vascular disturbances may propagate across physiological systems.

Endothelial dysfunction may also interact with several other domains:

- Immune activation may promote endothelial inflammation and vascular dysregulation (24, 25).
- Autonomic instability may alter vascular tone and hemodynamic responses (11, 12).
- Impaired perfusion may increase metabolic stress and mitochondrial strain (8).
- Endocrine signaling may influence vascular reactivity and nitric oxide availability (19).

These multidirectional interactions position endothelial dysfunction as an important regulatory node within the broader physiological network underlying Long COVID.

### 3.7 Gut dysbiosis, barrier dysfunction, and immune–metabolic coupling

Alterations in the gut microbiome have been reported in individuals with Long COVID and may contribute to persistent systemic symptoms in a subset of patients. Several studies have described changes in microbial composition following SARS-CoV-2 infection, including reductions in beneficial commensal organisms and enrichment of pro-inflammatory microbial taxa (14). Although the degree and persistence of these changes vary across cohorts, disruption of microbial homeostasis provides a biologically plausible mechanism linking gastrointestinal, immune, and metabolic disturbances.

The gastrointestinal tract plays a central role in immune regulation, metabolic signaling, and host–microbiome interactions. Disruption of microbial balance may therefore influence systemic inflammatory pathways and metabolic homeostasis. Microbial metabolites, including short-chain fatty acids, bile acid derivatives, and other signaling molecules, participate in host immune regulation and energy metabolism.

#### 3.7.1 Intestinal barrier dysfunction and microbial translocation

Beyond changes in microbial composition, disruption of intestinal barrier integrity has been proposed as a mechanism linking gut dysbiosis to systemic inflammation. Increased intestinal permeability may allow translocation of microbial components, including lipopolysaccharide (LPS), into systemic circulation, thereby promoting immune activation.

Markers associated with gut permeability and microbial translocation have been reported in some Long COVID cohorts (41–43). LPS exposure can stimulate inflammatory signaling pathways and endothelial activation, providing a plausible biological link between gut barrier dysfunction and broader systemic dysregulation.

Causality remains incompletely defined; the gut–immune interface represents a potential amplifier of systemic inflammation in chronic disease states. Similar mechanisms have been described in other conditions characterized by immune activation and metabolic dysfunction.

#### 3.7.2 Gut–brain and gut–autonomic interactions

The gut–brain axis represents a bidirectional communication network linking intestinal function, immune signaling, and central nervous system regulation. Neural, endocrine, and immune pathways connect the gastrointestinal tract with autonomic and central regulatory systems.

Alterations in gut microbiota may influence neurotransmitter synthesis, inflammatory signaling, and vagal afferent signaling pathways. Serotonin disruption has also been described in post-acute viral syndromes, including Long COVID, and may provide an additional gut–immune–neural link between altered intestinal signaling, vagal pathways, platelet biology, and neurocognitive or fatigue symptoms (47). Experimental and clinical studies have demonstrated that microbial metabolites can affect central nervous system function through immune and neurochemical signaling mechanisms (48, 49).

Gut dysbiosis may therefore contribute to symptoms such as fatigue, cognitive dysfunction, gastrointestinal disturbances, and autonomic instability through interactions along the gut–brain–immune axis.

Gut dysbiosis may also interact with several other physiological domains:

- Microbial translocation may amplify immune activation and inflammatory signaling (41–43).
- LPS-mediated signaling may influence endothelial function and vascular regulation (42, 43).
- Gut–brain signaling may interact with autonomic regulation (48, 49).
- Metabolic and inflammatory pathways may influence mitochondrial energy regulation (8, 24, 25).
- Gut-derived signaling may contribute to neuroinflammatory pathways, including kynurenine pathway activation (50–52).

These interactions illustrate how disturbances in gastrointestinal homeostasis may propagate across physiological systems and contribute to persistent symptom expression within a network-based model of Long COVID.

### 3.8 Mast cell activation and histamine-mediated signaling

Mast cells are immune cells located at mucosal and vascular interfaces where they play an important role in host defense, inflammatory signaling, and regulation of vascular permeability. Through release of mediators such as histamine, tryptase, prostaglandins, and leukotrienes, mast cells influence vascular tone, immune cell recruitment, and neural signaling.

Several investigators have proposed that mast cell activation may contribute to symptom patterns observed in subsets of individuals with Long COVID. Reported symptoms potentially consistent with mast cell mediator release include flushing, pruritus, urticaria, tachycardia, gastrointestinal disturbances, headaches, and episodic multisystem symptom flares (15).

Observational studies have reported mast cell–related mediator abnormalities or symptom patterns compatible with mast cell activation in some individuals following SARS-CoV-2 infection (15). In addition, some clinical reports have described symptomatic improvement or mast cell–stabilizing therapies in selected patients (17).

However, findings across studies are heterogeneous. Not all investigations have demonstrated consistent elevations in mast cell mediators, and the prevalence of mast cell–related abnormalities in Long COVID populations remains uncertain (16). As a result, mast cell activation is therefore interpreted as a potential contributing mechanism in subsets of patients rather than a universal feature of the condition.

#### 3.8.1 Histamine signaling and multisystem symptoms

Histamine is a biologically active amine that influences vascular permeability, smooth muscle tone, gastric secretion, and neural signaling. Histamine receptors are widely distributed across multiple organ systems, including the central nervous system, cardiovascular system, gastrointestinal tract, and skin.

Because histamine signaling can affect diverse physiological processes, dysregulated mast cell activity could theoretically contribute to multisystem symptoms observed in Long COVID. Histamine-mediated vasodilation may influence vascular tone, while histamine signaling within the central nervous system may affect sleep regulation, cognitive function, and sensory processing.

Histamine signaling may also interact with autonomic regulation. For example, histamine-mediated vasodilation can influence heart rate and blood pressure responses, potentially interacting with dysautonomia in susceptible individuals.

#### 3.8.2 Immune and neuroimmune interactions

Mast cells interact extensively with immune and nervous system signaling pathways. Mast cell mediators can influence cytokine production, endothelial permeability, and neural signaling. Conversely, inflammatory cytokines and neuropeptides may activate mast cells, creating bidirectional signaling loops.

In Long COVID, persistent immune activation described in some cohorts may therefore influence mast cell activity through inflammatory signaling pathways (24, 25). Mast cell mediators may in turn amplify inflammatory and vascular responses, potentially reinforcing physiological feedback loops.

Mast cell signaling may also interact with several physiological domains:

- Mast cell mediators may influence vascular permeability and endothelial signaling.
- Histamine signaling may interact with autonomic regulation and cardiovascular responses.
- Inflammatory signaling may activate mast cells through immune pathways (24, 25).
- Gastrointestinal mast cells may influence gut barrier function and microbiome interactions.

These multidirectional interactions position mast cell signaling as an amplifying domain that may amplify vascular, autonomic, and inflammatory dysregulation in susceptible subsets of patients.

### 3.9 Neuroendocrine and sex hormone dysregulation

Endocrine signaling regulates multiple physiological systems including metabolism, immune function, vascular regulation, and stress responses. Disruptions in hormonal signaling have been reported following acute SARS-CoV-2 infection and may contribute to persistent symptoms in subsets of individuals with Long COVID. Endocrine disturbances can be objectively assessed through hormonal testing and are conceptually targetable through modulation of endocrine signaling pathways.

Observational studies have described alterations in several endocrine axes following COVID-19 infection, including thyroid function, gonadal hormones, and stress-axis signaling (19–23). Although the magnitude and persistence of these abnormalities vary across studies, endocrine perturbations may influence multiple interconnected physiological systems.

Hormones play key roles in regulating mitochondrial metabolism, immune responses, and cardiovascular function. Even modest alterations in endocrine signaling may therefore influence energy metabolism, inflammatory pathways, and vascular regulation.

Neuroendocrine dysregulation may also interact with multiple physiological domains, including autonomic regulation, mitochondrial function, vascular signaling, and immune modulation. Hormonal perturbations may therefore contribute both to symptom persistence and to amplification of cross-system instability in Long COVID.

#### 3.9.1 Thyroid axis disturbances

Thyroid hormones regulate basal metabolic rate, mitochondrial activity, and oxygen utilization. Alterations in thyroid function have been reported following SARS-CoV-2 infection, including patterns suggestive of thyroiditis, transient hypothyroidism, and dysregulated thyroid hormone signaling (19, 22, 23).

Thyroid hormone abnormalities can influence fatigue, thermoregulation, cardiovascular function, and cognitive performance. Because thyroid signaling interacts with mitochondrial metabolism and autonomic regulation, disruptions in thyroid hormone balance may amplify symptoms in susceptible individuals.

However, thyroid abnormalities in Long COVID cohorts are not universal, and many patients demonstrate normal conventional thyroid laboratory values. Consequently, thyroid dysfunction likely represents a contributing factor in subsets of patients rather than a universal driver of persistent symptoms.

#### 3.9.2 Gonadal hormone alterations

Sex hormones influence immune function, vascular biology, and metabolic signaling. Observational studies have reported alterations in testosterone levels in men following SARS-CoV-2 infection (20, 21). Reduced testosterone has been associated with fatigue, decreased exercise tolerance, and changes in body composition.

Estrogen and progesterone also influence vascular function, inflammatory signaling, and autonomic regulation. Hormonal fluctuations may therefore affect symptom expression in women following infection.

As with thyroid findings, endocrine abnormalities appear heterogeneous across cohorts. The presence and persistence of hormonal disturbances likely vary among individuals depending on disease severity, baseline endocrine status, and recovery trajectory.

#### 3.9.3 Stress-axis and neuroendocrine regulation

The hypothalamic–pituitary–adrenal (HPA) axis regulates physiological responses to stress and interacts closely with immune and autonomic signaling pathways. Inflammatory cytokines can influence hypothalamic signaling and glucocorticoid regulation, while stress hormones can modulate immune responses.

Alterations in stress-axis signaling have been proposed as a potential contributor to fatigue and autonomic symptoms in post-infectious syndromes (19). Dysregulation of circadian hormonal rhythms could theoretically influence sleep architecture, immune signaling, and metabolic homeostasis.

However, available data remain limited and heterogeneous, and further research is needed to clarify the role of HPA-axis disturbances in Long COVID.

Within the network model, endocrine dysregulation may interact with several other domains:

- Thyroid hormones influence mitochondrial energy metabolism.
- Glucocorticoids regulate immune and inflammatory responses.
- Sex hormones influence vascular tone and endothelial function.
- Endocrine signaling interacts with autonomic regulation through stress-axis pathways.

These multidirectional interactions position endocrine signaling as a regulatory domain capable of influencing multiple physiological systems within the proposed Long COVID network framework.

## 4 Immune activation, persistence, and amplification loops

### 4.1 Persistent immune activation and inflammatory signaling

Multiple studies have reported immune perturbations in subsets of individuals with Long COVID, including altered cytokine profiles, T cell activation patterns, and shifts in innate immune signaling (24, 25). These findings are heterogeneous across cohorts, with variability in specific markers, magnitude, and persistence. Nonetheless, converging evidence suggests that sustained immune activation may be present in a proportion of patients.

Immune activation does not inherently imply ongoing systemic viral replication. Rather, it may reflect residual antigen exposure, tissue-restricted viral remnants, post-infectious immune recalibration, or secondary amplification arising from other domains such as gut barrier dysfunction or mitochondrial stress. In selected biologically defined subsets, persistent antigenic stimulation or immune dysregulation may function as a primary driver; however, current evidence does not support uniform immune activation across all Long COVID populations.

Inflammatory cytokines influence endothelial integrity, autonomic regulation, and mitochondrial metabolism (24, 25). Elevated inflammatory tone may reduce nitric oxide bioavailability, impair oxidative phosphorylation efficiency, and alter neuroendocrine signaling. Disruption of vagally mediated cholinergic anti-inflammatory signaling has also been proposed as a potential contributor to sustained inflammatory tone and autonomic imbalance in post-COVID states (53). Impaired cholinergic modulation may function as a mechanistic bridge linking immune activation and dysautonomia rather than representing an independent primary domain.

Small fiber neuropathy affecting sensory and autonomic fibers has also been reported in subsets of individuals following SARS-CoV-2 infection and may represent a structural manifestation of immune-mediated peripheral nerve injury rather than an independent upstream mechanism (31).

These interactions position immune activation as a dynamic amplifier within Long COVID pathophysiology. While it may predominate in certain enriched subsets, it is unlikely to represent a universally dominant mechanism across heterogeneous Long COVID cohorts.

Importantly, not all Long COVID cohorts demonstrate uniform inflammatory elevation. Some investigations report modest or inconsistent differences compared with controls, reinforcing the concept of biological heterogeneity (24, 25). In this model, immune activation is conceptualized as a context-dependent process whose role varies across individuals and over time.

### 4.2 Viral antigen persistence without established replication

Detection of SARS-CoV-2 RNA or antigen in tissue compartments months after acute infection has been reported in selected studies (26, 27). These findings raise the possibility that viral material may persist in certain anatomical reservoirs, including gastrointestinal tissue, in a subset of individuals.

However, detection of viral RNA or protein does not necessarily demonstrate active replication. Viral remnants may persist in a non-replicative form and may or may not be immunologically active. Current evidence does not uniformly support widespread, ongoing systemic viral replication in the majority of Long COVID patients.

In this context, viral persistence is conceptualized as a potential amplifier that could sustain localized immune activation in certain individuals rather than as a universal mechanistic explanation. The degree to which persistent viral material is causally linked to symptom generation remains under investigation.

### 4.3 Autoantibodies and receptor-level modulation

Autoantibodies have been reported in subsets of individuals with Long COVID, including antibodies targeting G protein–coupled receptors and other regulatory targets (28). The presence and functional significance of these antibodies vary across studies.

Autoantibody-mediated receptor modulation provides a biologically plausible mechanism for sustained dysregulation of autonomic, vascular, or endocrine signaling in selected individuals. However, prevalence estimates and reproducibility remain variable, and causality has not been conclusively established.

Within this framework, autoantibody formation is positioned as a secondary amplifying mechanism that may modify receptor responsiveness and reinforce domain-specific instability in biologically enriched subsets.

### 4.4 Neuroinflammation and central signaling alterations

Neuroinflammatory processes have been proposed in Long COVID based on imaging findings, cerebrospinal fluid studies, and experimental models (29, 30). Experimental evidence demonstrates that even mild respiratory SARS-CoV-2 infection can induce neuroimmune changes and myelin dysregulation in animal models (30).

While direct *in vivo* evidence in humans remains evolving, these findings support biological plausibility for central immune activation contributing to cognitive dysfunction, fatigue, and autonomic dysregulation in subsets of patients.

Neuroinflammation may interact bidirectionally with autonomic instability, mitochondrial dysfunction, and endocrine dysregulation. Central immune signaling alterations could function as an amplifier of multisystem symptom persistence.

### 4.5 Sleep disruption and circadian destabilization

Sleep disturbance is frequently reported in Long COVID, including insomnia, non-restorative sleep, altered sleep architecture, and circadian rhythm disruption (1, 25). Although sleep instability is unlikely to represent an initiating primary domain, it may function as a cross-domain amplifier within the broader physiological system.

Impaired slow-wave sleep and circadian misalignment can alter autonomic tone, increase pro-inflammatory cytokine signaling, disrupt glymphatic clearance, and impair mitochondrial recovery dynamics. Experimental models demonstrate that sleep restriction elevates inflammatory mediators and reduces vagal tone, providing biological plausibility for bidirectional interaction between sleep instability and immune–autonomic regulation.

Within this framework, sleep disruption is conceptualized as a destabilizing load factor that may lower the threshold for symptom exacerbation across multiple domains. Persistent sleep fragmentation may amplify bioenergetic inefficiency, worsen orthostatic intolerance, and reinforce stress-axis dysregulation. Accordingly, sleep instability may sustain network persistence even when primary initiating drivers have partially attenuated.

### 4.6 Immune–metabolic–vascular amplification loops

Immune activation can interact dynamically with other domains:

- Cytokine signaling may impair endothelial nitric oxide bioavailability and vascular reactivity (24, 25).
- Inflammatory mediators may alter mitochondrial efficiency and promote oxidative stress (24, 25, 50, 51).
- Gut-derived endotoxemia may sustain systemic immune activation (41–43).
- Neuroimmune signaling may influence autonomic tone and stress-axis regulation.

These bidirectional interactions create feedback loops capable of sustaining symptom expression even in the absence of active viral replication. Such loops are consistent with network medicine principles in which interacting regulatory nodes maintain chronic illness states (32).

This model does not exclude viral persistence, autoimmunity, or immune dysregulation as contributors. Rather, it positions them within a broader systems framework in which amplification, coupling, and feedback may be as important as the initiating trigger (Table 2).

**Table 2:** Examples of cross-domain coupling.

Primary domain	Example secondary interaction	Mechanistic consequence
Dysautonomia	Immune activation	Sympathetic-mediated inflammatory amplification
Mitochondrial dysfunction	Endothelial dysfunction	Impaired oxygen delivery and oxidative stress
Gut dysbiosis	Immune activation	LPS-mediated cytokine signaling
Mast cell activation	Autonomic instability	Histamine-driven tachycardia
Neuroendocrine dysregulation	Mitochondrial dysfunction	Altered metabolic substrate utilization

## 5 Translational implications and trial enrichment strategy

### 5.1 From symptom clusters to mechanism-enriched cohorts

The heterogeneity observed in Long COVID symptom profiles and treatment responses suggests that broad symptom-defined enrollment strategies may combine biologically distinct subgroups within single therapeutic cohorts (3, 4, 9, 10, 33, 34). When mechanistic diversity is high, therapeutic signals targeting specific physiological pathways may be diluted.

A mechanism-anchored framework offers an alternative stratification strategy. Rather than grouping patients solely by symptom similarity, stratification could incorporate measurable biological markers corresponding to dominant mechanistic domains. Examples include:

- Autonomic testing for dysautonomia (11, 12).
- Metabolic or exertional assessments for bioenergetic impairment (13).
- Vascular function testing or laboratory markers for endothelial dysfunction (8).
- Microbiome profiling or permeability-associated markers for gut dysregulation (14, 41–43).
- Mast cell–associated mediator panels in clinically suggestive cases (15–18).
- Endocrine testing for thyroid, adrenal, or gonadal axis perturbation (19–23).

This approach also aligns with emerging discussion of “treatable traits” in Long COVID, in which mechanism-focused studies may eventually support more personalized management by linking specific therapeutic strategies to biologically or clinically identifiable patient subgroups (5).

Such enrichment strategies would not eliminate heterogeneity but may reduce cross-domain dilution within trials targeting specific biological pathways.

### 5.2 Trial variability and biological diversity

Interventional studies in Long COVID have demonstrated mixed or modest benefits across broadly defined populations (33, 34). Adaptive platform designs, such as STIMULATE-ICP, aim to evaluate multiple therapeutic strategies within heterogeneous cohorts (35). However, if mechanistic drivers vary substantially between individuals, response variability may persist despite improved trial design.

Biological enrichment may improve interpretability by aligning therapeutic targets with measurable physiological disturbances. For example:

- Autonomic-modulating therapies may be more effective in cohorts with demonstrable dysautonomia.
- Vascular-directed therapies may show clearer effects in individuals with measurable endothelial dysfunction.
- Immunomodulatory strategies may benefit subsets with objective immune activation signatures.

Such stratification mirrors approaches used in other heterogeneous chronic conditions in which endotype-driven treatment improved signal detection and therapeutic precision (36, 37).

Importantly, this framework does not imply that symptom-based trials are invalid. Rather, it suggests that mechanistic alignment may enhance statistical power and biological interpretability.

### 5.3 Clinical assessment implications

A mechanism-informed approach may influence clinical evaluation strategies in Long COVID. Comprehensive assessment could incorporate structured autonomic testing, endocrine evaluation, metabolic assessment, and vascular function testing in selected patients, guided by symptom patterns but anchored in objective measurement.

This does not imply that all patients require extensive testing. Rather, clinical prioritization may focus on identifying dominant physiological domains that plausibly explain multisystem symptom expression.

Integration of objective measures may also reduce reliance on purely subjective symptom reporting. While symptom reporting remains essential for patient-centered care, it may not fully capture underlying biological heterogeneity (Table 3).

**Table 3:** Trigger patterns and hypothesized mechanistic domains (hypothesis-generating).

Trigger	Domain(s) most likely implicated	Physiological rationale
Cold exposure	Endothelial/microvascular dysfunction; dysautonomia	Impaired vasoconstrictive regulation or abnormal vascular reactivity may exaggerate perfusion instability
Heat exposure	Dysautonomia; endothelial dysfunction	Heat-induced vasodilation increases orthostatic stress and may unmask autonomic instability
Standing (Orthostatic stress)	Dysautonomia	Autonomic reflex failure or exaggerated sympathetic activation may impair blood pressure and heart rate regulation
Supine intolerance	Dysautonomia; neuroendocrine dysregulation	Altered baroreflex signaling or fluid redistribution may provoke autonomic or stress-axis symptoms
Meals (general)	Gut Dysbiosis; mast cell activation; dysautonomia	Post-prandial blood flow shifts, enteric–autonomic coupling, and mediator release may amplify systemic symptoms
Carbohydrate-heavy meals	Gut dysbiosis; bioenergetic dysfunction	Rapid glucose flux may exacerbate metabolic instability or post-prandial inflammatory signaling
High-fiber meals	Gut dysbiosis	Fermentation shifts and microbial metabolite production may transiently alter immune and autonomic tone
Emotions/Stress	Neuroendocrine dysregulation; dysautonomia	HPA axis activation and sympathetic signaling may amplify inflammatory and autonomic instability
Physical exertion	Mitochondrial/bioenergetic dysfunction; endothelial dysfunction	Impaired oxidative phosphorylation, redox recovery, or oxygen delivery may precipitate delayed symptom exacerbation

Trigger–domain associations are hypothesis-generating and non-specific. Multiple domains may be simultaneously activated in individual patients. Prospective validation against objective physiological measures is required.

To operationalize trigger-informed stratification while maintaining biological caution, a prototype mechanism-oriented screening instrument is provided in Appendix 1. This unvalidated tool is intended solely for hypothesis generation and research enrichment and is not proposed as a diagnostic instrument.

### 5.4 Hypothesis generation and future directions

The proposed framework generates testable predictions:

- Patients stratified by dominant mechanistic domains may demonstrate differential treatment responsiveness.
- Multi-domain coupling may predict more refractory symptom patterns.
- Secondary amplifiers such as autoantibodies or antigen persistence may correlate with specific primary domain instability.

Future research may evaluate whether biologically enriched enrollment strategies improve therapeutic signal detection compared with symptom-only stratification. Assignment of dominant mechanistic domains is intended to reflect the relative weight of converging physiological abnormalities, objective testing patterns, and reproducible trigger-response relationships rather than categorical or mutually exclusive classification. Multiple domains may coexist within individual patients, with multidomain coupling potentially contributing to greater clinical complexity. The framework remains empirically testable because competing mechanistic hypotheses should demonstrate differential physiological signatures, biomarker enrichment patterns, and therapeutic responsiveness across biologically stratified cohorts.

This approach does not assume that all proposed domains are present in all individuals, nor does it assume that a single domain dominates universally. Rather, it provides a structured method for investigating heterogeneity in a condition characterized by multisystem complexity.

Domain-based enrichment strategies could reduce therapeutic signal dilution in heterogeneous cohorts and improve effect-size detection in randomized trials.

## 6 Discussion

This manuscript proposes a mechanism-anchored framework for interpreting Long COVID as a heterogeneous network disorder arising from interacting physiological systems. The novelty of the present framework does not lie in proposing network medicine, biological stratification, or endotype-driven modeling as entirely new concepts, as these approaches have been applied in other heterogeneous chronic diseases (32, 36, 37). Related computational studies have also applied network-based approaches to post-COVID sequelae, including single-cell network-based drug repositioning strategies for post-COVID pulmonary fibrosis (54).

Rather, the proposed contribution is the integration and operationalization of these principles within a structured Long COVID-specific model linking measurable physiology to translational stratification and hypothesis generation through distinction of primary mechanistic domains from secondary amplifying processes.

This framework builds on existing symptom-based and mechanism-focused approaches by integrating multiple interacting physiological domains rather than isolating individual mechanisms. In this context, heterogeneity in symptom presentation may reflect variation in dominant physiological nodes and the degree of cross-domain coupling rather than distinct disease entities.

A central implication of this model is that stratification based on measurable biological domains may identify more physiologically coherent subgroups than symptom-based approaches alone. This has potential relevance for clinical trial design, where mechanistic heterogeneity may contribute to variable or attenuated treatment responses in broadly defined cohorts. The proposed framework may also have implications for service organization and multidisciplinary care models. Because dominant physiological patterns may differ substantially between individuals, mechanism-informed stratification could eventually support referral pathways or clinical programs aligned with predominant biological domains rather than symptom-based grouping alone. Such approaches may improve integration between autonomic, immunologic, vascular, metabolic, endocrine, and rehabilitation-focused services within heterogeneous post-infectious illness populations.

The framework is intentionally testable. It predicts that biologically stratified cohorts will demonstrate differential therapeutic responsiveness compared with symptom-defined populations. Failure to observe such differences would challenge the validity of the model and support alternative explanatory frameworks.

Importantly, this model does not assume a single unifying mechanism for Long COVID. Rather, it accommodates multiple interacting processes, including immune, metabolic, vascular, autonomic, endocrine, and microbiome-related pathways, consistent with current evidence describing biological heterogeneity across cohorts.

## 7 Limitations and counterarguments

Several limitations and alternative interpretations warrant consideration.

### 7.1 Heterogeneity of evidence across cohorts

A central limitation in Long COVID research is variability in study populations, case definitions, methodologies, biomarker selection, and follow-up duration. Across cohorts, no proposed mechanistic domain demonstrates uniform presence or magnitude (8, 24, 25). For example, inflammatory markers are elevated in some studies but not others (24, 25). Mast cell–associated mediator signatures have been reported in selected cohorts yet not consistently reproduced (15–18). Similarly, endocrine abnormalities may resolve in many individuals while persisting in defined subsets (22, 23).

This variability complicates efforts to construct a single unifying biological model. The framework proposed here does not presume universal activation of any specific pathway. Instead, it interprets heterogeneity as an intrinsic feature of the condition, consistent with a multisystem network disorder in which different biological nodes may predominate across individuals and over time.

### 7.2 The possibility of a single dominant upstream driver

One alternative interpretation is that Long COVID may ultimately reflect a unifying upstream mechanism, such as persistent viral replication, viral antigen persistence, or autoimmune dysregulation, with downstream physiological disturbances representing secondary manifestations.

Persistent SARS-CoV-2 RNA has been identified in multiple tissue compartments months after infection in autopsy studies (26, 27). These findings do not establish ongoing replication-competent virus and may not generalize to ambulatory Long COVID populations. Broader analyses of post-acute infection syndromes suggest that viral antigen persistence may sustain immune activation even in the absence of replication-competent virus (55). In parallel, systematic review–level evidence supports the presence of diverse autoantibody signatures in subsets of patients with persistent symptoms (28).

From this perspective, dysautonomia, endothelial dysfunction, mitochondrial strain, gut dysbiosis, and neuroinflammation could represent downstream effects of a shared immune driver. However, heterogeneity in immune signatures, variability in detection of viral material, and inconsistent interventional responses across cohorts suggest that no single mechanism uniformly accounts for all cases (8, 24, 25, 33–35).

The present framework does not reject the possibility of a dominant upstream driver in certain individuals. Rather, it proposes that, at a population level, Long COVID appears biologically heterogeneous, with multiple interacting nodes contributing variably across subsets.

### 7.3 Viral replication hypothesis and interventional evidence

A critical distinction must be made between viral antigen persistence and persistent viral replication. Detection of SARS-CoV-2 RNA or protein in tissue does not necessarily establish the presence of replication-competent virus. Demonstration of ongoing replication generally requires evidence of viable virus, active transcriptional activity, or longitudinal viral evolution. To date, such findings have been most consistently documented in immunocompromised individuals with prolonged infection, whereas evidence of sustained systemic viral replication in immunocompetent populations with Long COVID remains limited.

Several studies have reported persistence of viral RNA or antigen in tissue compartments months after infection, including gastrointestinal and other anatomical reservoirs (26, 27). These observations suggest that viral material may persist in certain individuals and may contribute to ongoing immune stimulation or inflammatory signaling in defined subsets. However, detection of viral components alone does not establish whether such material remains replication competent or functionally pathogenic.

Interventional studies provide additional context but remain inconclusive. Randomized and platform trials evaluating antiviral therapies, including nirmatrelvir–ritonavir, in broadly defined post-acute cohorts have demonstrated mixed or modest effects on symptom improvement (33, 34). These findings may reflect several possibilities, including heterogeneity in underlying mechanisms, timing of treatment relative to disease course, or the presence of viral persistence only in biologically defined subgroups.

Accordingly, current evidence does not exclude the possibility that persistent viral reservoirs or replication may contribute to symptom persistence in some individuals. Within the framework proposed in this manuscript, viral persistence is therefore conceptualized as a potential contributor or amplifier within specific biological subsets rather than as a universal explanatory mechanism across heterogeneous Long COVID populations.

Future studies integrating tissue-level viral detection with mechanistic phenotyping and antiviral responsiveness will be required to clarify the role of viral persistence in post-acute disease.

### 7.4 Causality versus association

Many reported biological abnormalities in Long COVID are associative rather than definitively causal. For example, altered kynurenine metabolism (50), endothelial dysfunction (8), autonomic instability (11, 12), and endocrine shifts (20–23) may contribute to symptoms but could also arise secondary to systemic stress, deconditioning, or overlapping chronic disease processes.

The operational criteria outlined in Section 3 emphasize measurability and multisystem explanatory potential but do not establish temporal primacy. Longitudinal studies are required to clarify whether specific domain disturbances precede symptom persistence or emerge as secondary adaptations.

### 7.5 Measurement constraints and biomarker variability

Measurement methodologies vary substantially across studies. Heart rate variability metrics differ in acquisition protocols (11, 12). Microclot detection techniques lack full standardization (46). Mast cell mediators may fluctuate and be sensitive to timing (15–18). Cytokine profiles differ depending on assay sensitivity and sampling intervals (24, 25). Microbiome analysis lacks universally standardized clinical thresholds (14), and viral persistence detection remains technically complex and variably sensitive (26, 27).

These methodological differences contribute to inconsistent prevalence estimates and complicate cross-study comparison. Biological enrichment strategies must therefore rely on reproducible, standardized measures to be clinically useful.

### 7.6 Overlap between domains

The proposed domains are biologically overlapping rather than rigidly separable. Autonomic dysfunction may coexist with endothelial impairment. Mitochondrial dysfunction may result from inflammatory signaling. Endocrine dysregulation may follow chronic stress physiology. Mast cell activation may function as primary in some individuals and secondary in others.

This overlap is acknowledged and forms the basis of the network model described earlier (32). The framework does not assume compartmentalization but proposes that identifying dominant or contributory nodes may improve therapeutic alignment even when multi-domain involvement is present. The operational definition of primary domains therefore serves as a translational tool rather than a strict biological taxonomy.

### 7.7 Overlap with other post-infectious syndromes

Long COVID shares clinical and biological features with other post-infectious and chronic multisystem conditions, including dysautonomia syndromes, post-exertional fatigue states, mast cell–associated disorders, and autoimmune phenomena. Similar network-based interpretations have been proposed in other chronic illnesses (32).

This overlap raises the possibility that Long COVID may represent a trigger for dysregulation within pre-existing susceptibility networks rather than a wholly distinct disease entity. Such an interpretation is compatible with the framework proposed here and reinforces the need for mechanistic rather than purely symptom-based classification.

However, the present framework remains specifically anchored in Long COVID because it is derived from physiological abnormalities, epidemiologic patterns, and mechanistic observations reported following SARS-CoV-2 infection, including endothelial injury, autonomic dysfunction, persistent antigen detection, immune perturbation, exertion-related metabolic abnormalities, and post-COVID cohort-specific biological findings (8, 11–14, 19, 24–26, 28, 29, 31).

### 7.8 Summary

This manuscript proposes a structured interpretation of mechanistic heterogeneity in Long COVID. It does not claim that:

- All six primary domains are present in every patient.
- Viral persistence is absent or irrelevant.
- Immune activation is universal.
- Any single pathway dominates across cohorts.

Rather, it advances the hypothesis that interacting biological nodes sustain symptom persistence in subsets of individuals and that mechanistic enrichment may improve therapeutic precision.

## 8 Conclusion

Long COVID is increasingly recognized as a biologically heterogeneous, multisystem condition. Symptom-based phenotyping has improved epidemiologic characterization of this heterogeneity, but similarity in clinical presentation does not necessarily imply shared underlying pathophysiology. When therapeutic cohorts are defined solely by symptom expression, biologically distinct mechanisms may be grouped together, potentially diluting treatment effects and complicating interpretation of interventional studies.

This manuscript proposes a complementary mechanism-anchored framework centered on recurrent and biologically measurable domains: autonomic dysfunction, mitochondrial and bioenergetic impairment, endothelial and microvascular dysfunction, gut dysbiosis and barrier disruption, mast cell–mediated signaling, and neuroendocrine dysregulation. In this model, primary domains are defined operationally by objective measurability, the capacity to generate multisystem symptom expression, and potential modifiability. Secondary processes including immune activation, autoantibody formation, viral antigen persistence, and neuroinflammatory signaling are conceptualized as amplifying factors that may reinforce dysregulation across domains rather than serving as universal initiating drivers.

A potential advantage of mechanism-guided stratification lies in clinical trial design. Many interventional studies in Long COVID enroll heterogeneous populations defined primarily by symptom persistence rather than biological mechanism. Mechanism-based enrichment strategies, widely used in other heterogeneous diseases, may provide a framework for improving therapeutic signal detection by aligning treatment targets with measurable physiological abnormalities.

Future research should prospectively evaluate whether biologically enriched populations defined by objective physiological markers demonstrate differential therapeutic responsiveness compared with symptom-defined cohorts alone. If mechanism-guided stratification improves translational precision, it may provide a practical framework for advancing both clinical research and therapeutic development in Long COVID.

## Data Availability

The original contributions presented in the study are included in the article/Supplementary material, further inquiries can be directed to the corresponding author.
