# Supplementary material for: Long COVID as a network disorder: a mechanism-anchored framework for biological stratification and therapeutic targeting
Source: Front Med (Lausanne). 2026 May 29;13:1841690. doi: 10.3389/fmed.2026.1841690 (PMC13259708; doi:10.3389/fmed.2026.1841690)
Supplement: Supplementary file 1 [file Data_Sheet_1.docx]

**Appendix Box 1. Prototype Mechanism-Oriented Screening Instrument (Hypothesis-Generating, unvalidated)**

**Purpose**

This instrument is proposed as a hypothesis-generating screening tool to estimate predominant mechanistic domains in individuals with Long COVID and to prioritize objective physiological testing or mechanistically enriched trial design. It is not intended for diagnostic use and has not undergone prospective validation.

**Scoring**

Each item is rated on a 4-point scale:

0 = Never
1 = Occasionally
2 = Often
3 = Almost always

**A. Orthostatic Dysautonomia (POTS Phenotype)**

1. Standing still for 5–10 minutes reliably worsens lightheadedness, palpitations, or cognitive fog.
2. Heat exposure or hot showers consistently provoke symptom worsening.
3. Symptoms improve noticeably when lying down.

**B. Hyperadrenergic or Sympathetic Dominance Pattern**

1. Episodes of internal tremor, shakiness, or “adrenaline surges” occur without clear trigger.
2. Emotional stress produces rapid heart rate, chest tightness, or disproportionate symptom escalation.
3. You experience periods of feeling simultaneously exhausted and overstimulated (“wired but tired”).

**C. Bioenergetic Dysfunction With Post-Exertional Malaise (PEM Pattern)**

1. Physical or cognitive exertion results in delayed symptom worsening 12–72 hours later.
2. Following overexertion, recovery requires more than 24 hours and may extend for several days.
3. Exertion lowers your functional baseline for several days.

**D. Bioenergetic Dysfunction Without Delayed Exacerbation**

1. Persistent low energy is present most days and does not fluctuate dramatically with activity level.
2. Mild exertion produces early muscle heaviness or rapid fatigue without delayed crash.

**E. Gut Dysbiosis or Barrier Dysfunction Pattern**

1. Meals in general trigger worsening fatigue, cognitive symptoms, or tachycardia within 1–3 hours.
2. Carbohydrate-rich meals worsen symptoms more than protein-based meals.
3. High-fiber meals or certain foods provoke bloating, abdominal discomfort, or systemic symptom flares.

**F. Mast Cell or Histamine-Mediated Pattern**

1. Foods or environmental exposures provoke flushing, itching, urticaria-like reactions, nasal symptoms, or palpitations.
2. Symptoms occur in episodic multi-system flares involving skin, gastrointestinal, and cardiovascular features.

**G. Neuroendocrine Dysregulation**

1. Since SARS-CoV-2 infection, you have experienced new hormone-related changes such as menstrual irregularity, reduced libido, erectile changes, or altered sexual function.
2. You experience persistent cold intolerance, slowed thinking, constipation, hair thinning, or dry skin not present prior to illness.
3. You experience abnormal stress tolerance, including exaggerated fatigue or crash after emotional stress.
4. Sleep is non-restorative even after adequate duration, and poor sleep reliably worsens symptoms the following day.

**Interpretation Framework (Hypothesis-Generating)**

This appendix presents a simplified illustrative prototype intended to demonstrate how symptom-trigger patterns may be organized into mechanistically relevant domains. A more detailed trigger–timing operational framework is presented separately in a companion manuscript focused on mechanistic phenotyping and clinical application.

Domain grouping is intended to illustrate how symptom-trigger patterns may be organized for hypothesis generation. Higher scores within a domain may suggest increased likelihood of involvement, but domains are not mutually exclusive and should not be interpreted as discrete diagnostic categories. Individual items may map to more than one biological mechanism, and the same symptom trigger may arise through different physiological pathways in different patients.

Sleep-related disruption may function as a cross-domain amplifier rather than an independent initiating mechanism. Final domain attribution requires clinical synthesis together with objective physiological, laboratory, or imaging measures. This prototype is therefore best understood as an illustrative research-enrichment tool rather than a validated questionnaire or clinical scoring instrument.

**Important**

This instrument is an unvalidated prototype intended for research hypothesis generation and mechanistic enrichment strategies. It is not a diagnostic tool. Domain attribution requires confirmation through objective physiological, laboratory, or imaging measures. Individual questionnaire items are not assumed to map uniquely to a single biological domain. Many symptom triggers may arise from multiple interacting mechanisms. Domain grouping is therefore intended as a probabilistic signal for hypothesis generation rather than a deterministic classification.
